# Analyzing the therapeutic effects of short wrist splint in patients with carpal tunnel syndrome (CTS) under ibuprofen treatment from an EMG-NCV perspective

**Published:** 2015

**Authors:** H Riasi, A Rajabpour Sanati, F Salehi, H Salehian, K Ghaemi

**Affiliations:** * Department of Neuroradiology, University of Zurich, Zurich, Switzerland; ** Medical Faculty, Birjand University of Medical Sciences, Birjand, Iran; *** Department of Pediatrics, Birjand University of Medical Sciences, Birjand, Iran; **** Department of Neurosurgery, Birjand University of Medical Sciences, Birjand, Iran

**Keywords:** Carpal Tunnel Syndrome, wrist splint, NSAIDs, treatment

## Abstract

**Objective:** Carpal tunnel symptoms are typical outside nerve involvement and a significant reason of inability. This syndrome often causes weakness and pain in the affected hand. Therefore, the researchers decided to perform a study that aimed to investigate the therapeutic effects of Wrist Splint (short wrist splint) in patients admitted to the neurology clinic of Vali Asr Hospital of Birjand with carpal tunnel syndrome (CTS) under ibuprofen treatment from an EMG-NCV perspective.

**Methods:** This clinical trial was performed on patients admitted to the neurology clinic of Vali Asr Hospital during the spring and summer of 2013. The participants of the study were classified into two teams. The initial team of cases went under medical treatment with ibuprofen (800 mg BID for 4-6 weeks) and the second group of patients went under medical care with ibuprofen (800 mg BID for 4-6 weeks) with a short wrist splint.

**Results:** 40 cases participated in the research. The mean age of participants was 32/ 75 ± 4/ 33 years (range 20 to 48 years), respectively. Most of the participants were females (n = 30, 75% respectively). The mean score, latency and velocity between the two groups were mainly distinct (respectively p = 0.05, 0.001, and 0.003). There were significant differences between amplitude before the start of the study (p = 0.000) among the participants.

**Discussion:** The results showed that patients treated with ibuprofen and wrist splint along the para-clinics presented a better response to treatment.

## Introduction

CTS is the most typical syndrome of peripheral nerve involvement and the primary reason of inability [**1**]. Carpal tunnel is limited posteriorly by the carpal bones, and anteriorly by the transverse wrist ligament. Such anatomical constraints and the pressure caused by the repeated application of the extensor and flexor muscles passing through the carpal tunnel, prepares the means to the most common compression neuropathy, the carpal tunnel syndrome [**2**]. Various predisposing factors may be held responsible for the formation of CTS. The epidemiology of this disease mainly consists of otherwise healthy women of over 40 years. This syndrome is mainly caused by the compression of the mean nerve, which can be due to a first reduction in the dimension of the canal itself, or a subsequent decrease caused by the enlargement of its contents, thus leading to the accommodation of more free space. Other risk factors include diabetes, hypothyroidism, fractures, and dislocations of the wrist and arthritis. Weakness or impaired use of the hands, numbness, and tingling and muscle damage are the most common symptoms experienced by CTS patients. The diagnosis, which is carried out by doctors, is mainly based on a detailed description of the clinical symptom and tests, like the Phalen’s maneuver and Tinel’s symptom. The severity of the condition is determined either by NCV or EMG clinical examinations [**3**]. Treatment is either surgical or non-surgical [**1**]. The initial choice of therapy is determined by different parameters like duration of symptoms, age, technical consideration, clinical results, EMG results, history of before treatments, and the possibility of technical modifications. Short wrist splints and the prescription of NSAIDs (Non-steroidal anti-inflammatory drugs) are two of the most typically employed therapeutic procedures [**2**]. Using short wrist braces, especially during nighttime, helps stabilize the wrist and relieve pressure off the median nerve, also reducing symptoms, especially in light and moderate cases [**3**]. The duration required for wearing splints varies. However, braces are usually worn until the complete recession of the symptoms, which usually takes up to 4-6 weeks. NSAIDs and oral corticosteroids may be effective for a short time (2 to 4 weeks). However, the administration of local corticosteroids may be more efficient for long periods [**4**]. The prevalence of this disease is 2.9% in women and 0.6% in men [**2**]. Some of the signs of carpal tunnel syndrome include tingling in the fingers, numbness, pain in the thumbs originating from the wrist to the fingers, compromise in a tactile sense and temperature perception, and swelling of the hands and forearms [**5**]. Since numbness and tingling may be mild and occur only periodically, many patients do not seek medical attention. However, the disease may progress towards numbness and continuous irritation. In some chronic and severe cases, muscle atrophy occurs at the thenar surface (the surface covering the palmar side of the halluces) [**5**]. If symptoms prove to be resistant to conservative treatments, surgical procedures are usually taken into consideration [**6**].

In addition, the prescription of NSAIDs and short wrist splints are two primary traditional approaches in treating CTS patients. To discover a more efficient therapeutic procedure, we decided to compare the effect of NSAID administration and short wrist splints based on para-clinical criteria. Since the leading cause of carpal tunnel syndrome is substantial work and continuous use of the carpal joints, it was decided that the research would only consider these parameters, disregarding the effect of other factors.

## Methodology 

The current research was carried out as a clinical trial on patients admitted to the Neurology Clinic of Vali Asr Hospital of Birjand during spring and summer, 2013. A census sampling was performed, accepting all the patients who had visited the Neurology Clinic of Vali Asr Hospital and met the requirements of entering the study, as well as lacking exclusion parameters. Their condition must not have led to other complications, and no muscular atrophy should have been present. The patients were subjected to the study for a total duration of three months. Based on the similar period of the previous year, the subject population was estimated to consist of 50 patients. 

The statistical population consisted of referred and directly admitted patients who had visited the neurology clinic, with a chief complaint of neurologic symptoms. CTS diagnosis was confirmed based on clinical examinations (Phalen’s maneuver and Tinel’s sign) and a proximal and distal amplitude difference higher than 50% and delayed distal motor conduction velocity lower than 20 m/ s. The inclusion criteria were the following: 1- personal consent of the subjects. 2- patients must not be pregnant at the time of entering the study, and their condition must not be the result of maternity and pregnancy. 3- Subject’s carpal tunnel syndrome should not be a complication of tumors in the carpal region or a result of trauma/ fracture to the carpal bones. 4- Subject should not have suffered from the cellular damage of the upper limb or their spine before entering the study. 5- Subjects must not have any history of substance abuse. 6- No signs of denervation should come up in the EMG scan results of the patients. Also, their condition should not have led to other complications such as thenar atrophy. 7-Subject’s condition must not be the result of RA (rheumatoid arthritis) or other collagen vascular disorders. 8- The subjects condition must not be the result of metabolic disorders such as diabetes or disorders of the thyroid gland, 9- Subjects must be conscious to a degree which enables them to partake in follow-up studies and comply with their physicians’ necessary recommendations. Exclusion criteria included their absence in follow up re-examinations and clinical tests, and specific organic pathological disorders such as cancers, tumors, fractures, and collagen vascular diseases such as RA, metabolic diseases such as diabetes, thyroid disorders, or other problems that may be disruptive in the study process. 

The research was carried out according to the following procedures: The patients who were admitted to the Neurology Clinic of Vali Asr Hospital either as outpatients or by referrals from other doctors and specialists (e.g., internal medicine and orthopedic specialties) and their clinical symptoms were significant of carpal tunnel syndrome. Underwent physical examination and clinical tests, a neurologist confirming their condition as carpal tunnel syndrome. By providing the necessary information, their consent was obtained. If other inclusion criteria were also met, the initial results of the clinical tests, including NCV and EMG examinations, were recorded, and the participants were divided into two groups, each consisting of 25 subjects. 

The patients of the first group were only treated with ibuprofen (800 mg BID for 30-45 days), while the patients of the second group were treated with wrist splints in addition to ibuprofen administration (800 mg BID for 30-45 days). The necessary medical advice was given to both groups.

After 4 to 6 weeks of treatment, the patients were asked to readmit to the Neurology Clinic where they underwent the physician’s examination for the second time. Para-clinical tests (NCV and EMG) were also performed. The gathered results were once again recorded and analyzed to find any possible change in the condition of the disease. 

What should also be noted is that for most of the participants, routine EMG & NCV tests were both necessary.

The gathered data were recorded in the form of checklists, by entering them into an SPSS 18 software file. The data were excavated by using descriptive statistics (frequency percent and mean) as well as analytical statistics.

## Results

A whole of forty cases participated in this research. Their average age was 32.75 ± 4.33 years (ranging from 20 to 48 years). Most of the study participants were females (30 subjects, 75%). The majority of the members were homemakers (22 subjects, 55%). Most of the participants had a high school education level (20 subjects, 50%). **Tables 1** to **3** show the demographic frequency distribution of the subjects’ information.

No significant difference was present between the average age of the participants in the group treated solely with ibuprofen and the group receiving both ibuprofen and splinting treatment (P = 0.46). **Table 4** compares the median age in both groups. In this study, no significant difference was present considering the gender of the participants between the ones treated solely with ibuprofen and the group receiving both ibuprofen and splinting treatment (P = 0.23). **Table 5** represents a sex comparison between the two groups above. No significant difference was also present relevant to the technical considerations of the members of either of the groups (P = 0.87). **Table 6** represents a comparison of professions between the two groups. 

Before the study, the Average m latency, s latency and velocity were determined, being 8.36, 9.54 and 4.95 in the ibuprofen only treatment group and 8.49, 8.87 and 2.99 in the ibuprofen and splint treatment group (p = 0.56, p = 0.89, and p = 0.23, respectively). No significant difference was present between the participants considering the term of amplitude difference before the engagement in the study (p = 0.49). **Table 7** compares the average values of m latency, s latency, velocity, and amplitude difference before interventions.

A significant difference was present between the mean of s latency and velocity values after interventions between the group treated with only ibuprofen and the group treated with ibuprofen and splints (p = 0.05, p = 0.001, p = 0.03, respectively). A significant difference was also evident considering the amplitude difference after the study (P = 0.000). **Table 8** compares the average values of s latency, m latency, and velocity and amplitude difference after interventions. 

**Table 1:** Average age, age range, and age group of the study participants

	Frequency (percent)	Average	Interval
Age (N = 40)		32.75 ±4.33	20-48
20-30	14 (35)		
30-40	21 (52.5)		
40-50	5 (12.5)		

**Table 2:** Sex frequency distribution of the subjects

Sex (n = 40)	Frequency	Percent
Male	10	25
Female	30	75

**Table 3:** The profession frequency distribution of the subjects

Profession (N = 40)	Frequency	Percent
Household	22	55
Employee	12	30
Freelance	6	15

**Table 4:** Comparison of the average age between the group receiving ibuprofen and the group treated with ibuprofen and splints

	Average Age	P value
Group with Ibuprofen (n = 20)	30.23 ±1.24	P = 0.46 t = 0.234
Group with Ibuprofen + Splinting (n = 20)	31.15± 2.12	

**Table 5:** A sex comparison between the group receiving ibuprofen and the group treated with ibuprofen and splints

	Sex		
	Female Frequency (Percent)	Male Frequency (Percent)	P value
Group with Ibuprofen (n = 20)	6 (30)	14 (70)	P = 0.23 t = 0.178
Group with Ibuprofen + Splinting (n = 20)	4 (20)	16 (80)	

**Table 6:** A comparison of professions between the two relevant study groups

	PROFESSION			
	Household Frequency (Percent)	Employee Frequency (Percent)	Employee Frequency (Percent) Freelance Frequency (Percent)	P value
Ibuprofen (n = 20)	12 (60)	5 (25)	3 (15)	P = 0.87 X2 = 1.333
Ibuprofen + Splinting (n = 20)	10 (50)	7 (35)	3 (15)	

**Table 7:** A comparison between the average values of m latency, s latency, velocity, and the frequency distribution of amplitude difference prior to the intervention in the two study groups

	m latency	s latency	velocity	Amplitude Difference	
				Less than 50%	Less than 50%
Ibuprofen (n = 20)	8.36	9.54	4.95	5 (25)	15 (75)
Ibuprofen + Splinting (n = 20)	8.49	8.87	2.99	4 (20)	16 (80)
P value	P = 0.56 t = 0.357	P = 0.89 t = 0.705	P = 0.23 t = 0.593	P = 0.49 t = 0.432	

**Table 8:** A comparison between the average values of m latency, s latency, velocity, and the frequency distribution of amplitude difference subsequent to intervention in the two study groups

	m latency	s latency	velocity	Amplitude Difference	
				Less than 50%	Higher than 50%
Ibuprofen (n = 20)	4.40	5.10	12.18	15 (75)	5 (25)
Ibuprofen + Splinting (n = 20)	3.33	3.86	12.23	19 (95)	1 (5)
P value	P = 0.05 t = 3.393	P = 0.001 t = 2.658	P = 0.03 t = 3.996	P = 0.000 t = 7.658	

## Discussion

The current article aimed to evaluate the therapeutic effects of short wrist splints from an EMG/ NCV perspective on patients suffering from carpal tunnel syndrome undergoing medical treatment with ibuprofen. The results indicated that the group treated with ibuprofen and splints, showed better therapeutic results in a para-clinical manner regarding the clinical response. 

In the current research, a significant difference was present between the average values of m latency, s latency and velocity after the intervention in both groups undergoing examination (P = 0.05, 0.001, and 0.03, orderly). Also, a significant difference was evident between the participants regarding the amplitude difference after intervention (P = 0.000). 

In agreement with the findings of the present research, Necmettin Yildiz concluded that the use of anti-inflammatory drugs along with short wrist splints improves clinical symptoms in patients with carpal tunnel syndrome [**7**]. However, it should be noted that other than ibuprofen, many anti-inflammatory drugs are also useful in the treatment of this disease. For example, a study by Celiker and colleagues performed in 2002, aimed to compare the effectiveness of bounded corticosteroid injection with NSAID and splinting in treating the carpal tunnel symptom. The results showed that the simultaneous use of both therapeutic methods could lead to a significant improvement and showed similar effects in the patients with carpal tunnel syndrome [**10**]. Therefore, the most preferred procedure must be selected based on the patient’s clinical condition and the social and cultural requirements. 

Also consistent with the findings of this research, Hall indicated that the sole application of short wrist splints is proper in the improvement of the symptoms and functional status of the disease [**8**]. Inconsistent with the results of the current study, in a research performed by Gerritsen and his colleagues in 2003, aiming to evaluate the prognosis for long-term use of short wrist splints in patients with CTS, results showed that recovery was only evident in a limited number of the participants [**11**].

Although a clear improvement was seen in the para-clinical symptoms of the cases partaking in the research, it should be noted that these findings are only relevant to this study and only analyzing short-term effects. Therefore, the results may not be extended to long runs. A survey carried out by Sevier and Wilson showed that no steroidal anti-inflammatory drugs and injections and short wrist splinting were effective in reducing symptoms, but the results were poor on long term [**9**]. To the contrary, in another research performed by Gerritsen in 2002, aiming to evaluate the short-term and long-term effects of short wrist supporting and operation to relieve the signs of CTS, the outcomes showed a significant improvement in symptoms, on both short term and long-term follow-ups [**12**]. 
